# Transcriptomic analysis of *Clostridium thermocellum *ATCC 27405 cellulose fermentation

**DOI:** 10.1186/1471-2180-11-134

**Published:** 2011-06-14

**Authors:** Babu Raman, Catherine K McKeown, Miguel Rodriguez, Steven D Brown, Jonathan R Mielenz

**Affiliations:** 1 Biosciences Division, Oak Ridge National Laboratory, One Bethel Valley Road, Oak Ridge, TN 37831, USA; 2 BioEnergy Science Center (BESC), Oak Ridge National Laboratory, One Bethel Valley Road, Oak Ridge, TN 37831, USA; 3 Bioprocess R&D, Dow AgroSciences, 9330 Zionsville Road, Indianapolis, IN 46268, USA

## Abstract

**Background:**

The ability of C*lostridium thermocellum *ATCC 27405 wild-type strain to hydrolyze cellulose and ferment the degradation products directly to ethanol and other metabolic byproducts makes it an attractive candidate for consolidated bioprocessing of cellulosic biomass to biofuels. In this study, whole-genome microarrays were used to investigate the expression of *C. thermocellum *mRNA during growth on crystalline cellulose in controlled replicate batch fermentations.

**Results:**

A time-series analysis of gene expression revealed changes in transcript levels of ~40% of genes (~1300 out of 3198 ORFs encoded in the genome) during transition from early-exponential to late-stationary phase. K-means clustering of genes with statistically significant changes in transcript levels identified six distinct clusters of temporal expression. Broadly, genes involved in energy production, translation, glycolysis and amino acid, nucleotide and coenzyme metabolism displayed a decreasing trend in gene expression as cells entered stationary phase. In comparison, genes involved in cell structure and motility, chemotaxis, signal transduction and transcription showed an increasing trend in gene expression. Hierarchical clustering of cellulosome-related genes highlighted temporal changes in composition of this multi-enzyme complex during batch growth on crystalline cellulose, with increased expression of several genes encoding hydrolytic enzymes involved in degradation of non-cellulosic substrates in stationary phase.

**Conclusions:**

Overall, the results suggest that under low substrate availability, growth slows due to decreased metabolic potential and *C. thermocellum *alters its gene expression to (i) modulate the composition of cellulosomes that are released into the environment with an increased proportion of enzymes than can efficiently degrade plant polysaccharides other than cellulose, (ii) enhance signal transduction and chemotaxis mechanisms perhaps to sense the oligosaccharide hydrolysis products, and nutrient gradients generated through the action of cell-free cellulosomes and, (iii) increase cellular motility for potentially orienting the cells' movement towards positive environmental signals leading to nutrient sources. Such a coordinated cellular strategy would increase its chances of survival in natural ecosystems where feast and famine conditions are frequently encountered.

## Background

Among cellulolytic microorganisms, the anaerobic, thermophilic, Gram-positive bacterium, *Clostridium thermocellum *displays one of the fastest growth rates on crystalline cellulose [1,2]. This native cellulolytic organism encodes a repertoire of carbohydrate active enzymes (CAZymes) for degradation of plant cell wall polysaccharides, which are assembled in large enzyme complexes, termed cellulosomes, on the cell surface [3,4]. *C. thermocellum *is thus capable of both deconstructing crystalline cellulose into oligomeric cello-oligosaccharides and fermenting the hydrolysis products directly to ethanol and other organic acids, consequently minimizing or eliminating the need for external addition of non-native hydrolytic enzymes. Elimination of a separate cellulase-production step is economically advantageous for industrial cellulosic ethanol production processes [5,6]. *C. thermocellum *is therefore an attractive candidate microorganism for consolidated bioprocessing of lignocellulosic biomass to biofuels.

Several past studies have investigated the expression and regulatory nature of approximately two dozen selected genes encoding cellulosomal catalytic and structural components in *C. thermocellum *[7-12]. Dror *et al*. reported growth-rate dependent regulation of cellulosomal endoglucanases (*celB*, *celD*, *celG*) and the major processive endoglucanase *celS *[7,9]. A growth-rate dependent variation of mRNA levels was also reported for the cellulosome scaffoldin genes *cipA *and the anchor genes *olpB *and *orf2p *but not *sdbA *[8]. In continuous cultures studies, Zhang and Lynd, using an ELISA method, suggested cellulase synthesis in *C. thermocellum *to be regulated by a catabolite repression type mechanism [12]. Sparling, Levin and colleagues have investigated the gene expression and enzymatic activities of several proteins involved in pyruvate metabolism and fermentation [13,14].

A draft assembly of the *C. thermocellum *genome sequence became available in 2003, which was subsequently completed and the genome was closed in 2006. This paved the way for whole-genome gene and protein expression studies. We previously reported the construction and evaluation of a whole genome oligo-nucleotide microarray with probes representing ~95% of the open reading frames based on the draft assembly of the *C. thermocellum *genome sequence [15]. Microarrays are invaluable research tools that provide comprehensive information on the underlying molecular mechanisms for cellular behavior, states and transcriptional regulation. In this study, microarray technology was used to investigate the temporal changes in gene expression associated with fermentation of crystalline cellulose by *C. thermocellum*.

Overall, the gene expression patterns revealed a coordinated response by *C. thermocellum *to conditions of altering substrate availability during cellulose batch fermentations. *C. thermocellum *modulates the composition of cellulosomes released into the environment in stationary phase and enhances signal transduction, chemotaxis mechanisms probably for sensing of substrate gradients resulting from the action of cell-free cellulosomes. *C. thermocellum *also increases expression of genes involved in cellular motility function, potentially to orient the movement of cells towards available nutrient sources in the environment. Such a coordinated cellular strategy should increase its chances of survival under conditions akin to feast and famine that are frequently encountered in natural ecosystems. To our knowledge, this is the first study looking at the transcriptional response of *C. thermocellum *at a global level and provides the foundation for future research using natural biomass as growth substrates.

## Methods

### Fermentation

*C. thermocellum *ATCC 27405 wild-type strain was a gift from Prof. Herb Strobel at the University of Kentucky, Lexington, KY. Batch fermentations were conducted in 3 L BioStat B jacketed glass fermentors (Sartorius Stedim Biotech, Bohemia, NY) using a 2 L working volume of MTC medium (mineral salt medium containing 1 g/L yeast extract; [16]) at 58°C and 300 rpm, with pH controlled at 7.0 using 3N NaOH. Fermentors with medium containing only the carbon substrate, 5 g/L crystalline cellulose (Avicel^® ^PH105, FMC Biopolymer, Philadelphia, PA), were sparged with ultra-high purity nitrogen and vigorously agitated overnight, followed by addition of the remaining medium components and sparged for an additional 2-3 hrs with nitrogen gas. A 10% v/v inoculum of overnight (16-20 hrs) 5 g/L Avicel^® ^bottle cultures was used to inoculate the fermentors and the gas inlet/exhaust lines were clamped post inoculation.

### Protein and metabolite analysis

Well-mixed 2 mL aliquots of cultures were harvested at regular intervals and centrifuged quickly to separate into pellet and supernatant samples for protein analysis of pellet fractions and HPLC analysis of extracellular metabolites, respectively. Cell growth was monitored based on increase in protein content within the total solids present in the pellet fraction, including the Avicel^® ^substrate [16]. Briefly, the solid pellet was washed with de-ionized water and the cells were lysed using 0.2N NaOH/1% w/v SDS solution, cell debris were pelleted and removed, and protein concentration in the clear supernatant was estimated using the bicinchoninic acid protein assay (Pierce Chemical, Rockford, IL). Metabolite analysis was performed using a LaChrom Elite system (Hitachi High Technologies America, Inc., Pleasanton, CA) equipped with a refractive index detector (Model L-2490). Metabolites were separated at a flow rate of 0.5 mL/min in 5 mM H_2_SO_4 _using an Aminex HPX-87H column (Bio-Rad Laboratories, Inc., Hercules, CA).

### RNA isolation and microarray analysis

Fermentation samples for RNA isolation were harvested by spinning down ~30 mL culture in 50 mL Oak Ridge tubes at 8000 rpm and 4°C for 10-15 mins and the supernatant was discarded. The solid pellet fraction containing cells and any residual Avicel^® ^was resuspended in 1 mL of TRIzol (Invitrogen, Carlsbad, CA), flash frozen in liquid nitrogen and stored at -80°C until further use. Total RNA was extracted from the cell pellets as follows. Briefly, the frozen cell solution in TRIzol was thawed on ice and the cell solution (~1 mL) was added to a 2 mL tube containing 1 mL of 0.1 mm glass beads (BioSpec Products, Bartlesville, OK) ashed at 250°C overnight. Cells were lysed by rapid agitation of the tubes at 6500 rpm for 1 min in three 20s-On/20s-Off cycles using the Precellys^® ^bead beater (Bertin Technologies, France). Subsequently, the cell lysate (~0.8 mL) in TRIzol was phase separated by addition of 200 μL chloroform and the RNA was precipitated by addition of 500 μL 100% isopropanol. The precipitated RNA pellet was washed with 1 mL of 75% ethanol and resuspended in 100 μL of RNase-free water. Any contaminating DNA was digested by in-solution DNase-I (Qiagen, Valencia, CA) treatment and the RNA sample was cleaned using the RNeasy mini kit (Qiagen, Valencia, CA) as per manufacturer's instructions. The 6 hr time-point RNA sample was used as the reference and all other time-point samples (8, 10, 12, 14, 16 hr) were compared to the reference in cDNA/cDNA arrays. For each time-point comparison, equal amount of the extracted total RNA samples was labeled with Cy3-dUTP/Cy5-dUTP fluorescent dyes (GE Healthcare, Piscataway, NJ), mixed and hybridized onto custom oligo-arrays in dye swap experiments as described earlier [17] and microarray slides were scanned in ScanArray Express scanner (Perkin Elmer, Waltham, MA).

### Microarray construction and statistical data analysis

Microarrays containing 2980 unique and 10 group 70-mer oligonucleotide probes representing ~97% of the 3163 Open Reading Frames (ORFs) in the draft assembly of *C. thermocellum *ATCC 27405 were constructed as described earlier [15]. The probe sequences were later compared to the completed genome sequence using reciprocal BLAST analysis and assigned new ORF numbers. Based on the comparison, 79 probes which did not have any BLAST hits and 108 probes that only had partial hits to annotated ORFs in the closed genome were either excluded or marked-up during downstream data analysis. Signals were quantified in ImaGene version 6.0 (BioDiscovery Inc., El Segundo, CA) and statistical data analysis was conducted using JMP Genomics software (SAS Institute Inc., Cary, NC). The array signal intensities were background-corrected, log_2_-transformed and data for duplicated probes on the arrays were averaged and normalized using the Data-Standardize method. Low-quality array data were discarded based on scatter plots, correlation coefficients, principal component analysis and other quality control criteria. One-way ANOVA analysis was conducted with the 6 hr samples as the control at a False Discovery Rate of 2% (P-value < 0.01) to identify differentially expressed genes of statistical significance. Genes significantly up- or down-regulated in at least one time-point comparison were analyzed in TIGR MeV 4.5 software [18] to identify similar temporal trends in gene expression using average-linkage hierarchical or K-means clustering methods.

## Results and Discussion

**Figure 1:**
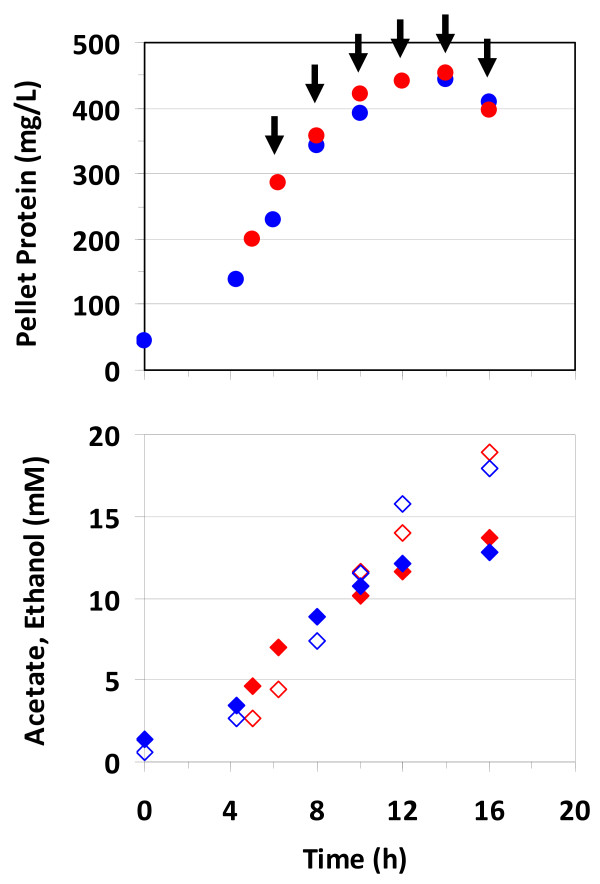
**Fermentation growth and metabolite production plots**. Pellet protein-based growth and metabolite curves for duplicate *Clostridium thermocellum *ATCC 27405 fermentations on 5 g/L crystalline cellulose (Avicel^®^). Arrows in the upper panel indicate culture sampling points for microarray-based gene expression analysis. Acetate and ethanol data in the lower panel are shown in closed and open symbols, respectively.

In this study, we investigated the global changes in gene expression associated with fermentation of crystalline cellulose by the anaerobic bacterium *Clostridium thermocellum*. In order to achieve this, we conducted duplicate 2 L batch fermentations of *C. thermocellum *on 5 g/L of crystalline cellulose (Avicel^®^) and took a series of six time-point samples ranging from early-exponential to late-stationary phase of cell growth. Cell growth was monitored based on total cellular protein content in the solid pellet fraction which continued to increase until ~12 h when cells entered stationary phase (Figure 1). No visible residual Avicel^® ^was found at the end of the fermentation. Metabolite analysis revealed an inversion of acetate-to-ethanol molar ratios over the course of the fermentation, with higher molar levels of acetate than ethanol in the beginning of the fermentation, but the ratio decreased to ~0.7 towards the end of the fermentation (Figure 1). Unlike earlier reports, no detectable levels of lactate or formate were identified in the fermentations, possibly due to differences in culture conditions. For instance, while lactate and formate were readily detected in batch experiments using Balch tubes with no pH control [19,20], they were formed at very low rates in controlled fermentations in bioreactors with pH control [21]. Moreover, these metabolites may not have been detected in this study possibly due to differences in the detection method (refractive index *vs *conductivity detector) used in HPLC measurements.

Total RNA was extracted from the cell pellets and the reverse transcribed cDNA was hybridized to oligo-arrays containing duplicated probes representing ~90% of the annotated ORFs in *C. thermocellum *ATCC27405 genome. Dual-channel dye swap experimental design was used to analyze the time-course of gene expression during cellulose fermentation using the 6 hr sample as the reference, to which all other samples were compared. The entire microarray dataset has been deposited in NCBI's Gene Expression Omnibus (GEO, [22]) database under accession number GSE29554 (http://www.ncbi.nlm.nih.gov/geo/query/acc.cgi?acc=GSE29554). Data analysis revealed over ~1300 genes that were differentially expressed with statistical significance in at least one time point comparison. This represents ~40% of 3198 ORFs in *C. thermocellum *showing significant changes in gene expression over the course of cellulose fermentation. Gene expression ratios estimated by microarray methods displayed high correlation with those measured by quantitative RT-PCR, for five representative genes across two different time-points, with an R-value of 0.92 (Additional file 1). Hierarchical clustering and principal component analysis of sample datasets revealed clustering of the 6 h exponential sample distinctly from the rest of the time points. Among these were three branches corresponding to late exponential phase (8, 10 h), transition to stationary phase at 12 h and late stationary phase samples (14, 16 h) (data not shown).

**Figure 2:**
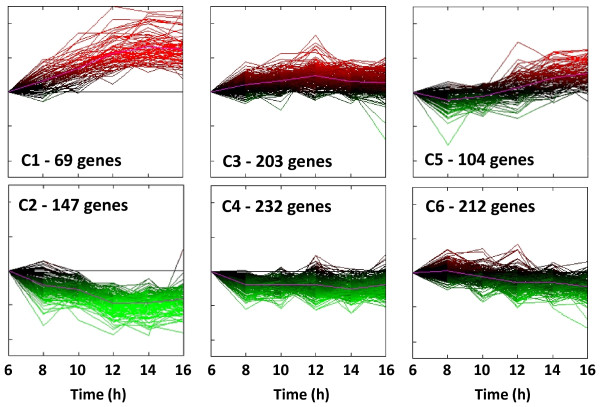
**Temporal expression-based clustering of genes differentially expressed during cellulose fermentation**. K-means clustering of genes that were differentially expressed in time-course analysis of transcript level changes during Avicel^® ^fermentation by *Clostridium thermocellum *ATCC 27405. Total of 967 genes (excluding 321 genes encoding hypothetical and proteins of unknown function) were clustered into 6 bins based on Euclidean distance using the TIGR MeV^® ^4.0 software.

K-means clustering algorithms were used to group the 967 differentially expressed genes (Additional file 2), excluding 321 genes encoding hypothetical and proteins of unknown function (Additional file 3), into six distinct clusters based on the similarity of their temporal expression profiles (Figure 2). The six clusters broadly represented mirror-images of three different temporal patterns in gene expression, namely (i) genes which show significant continually increasing or decreasing trends in expression over the entire course of the fermentation (Clusters C1 and C2, respectively), (ii) genes which show a moderate increase or decrease in expression during exponential growth until reaching stationary phase around 12 h but do not change thereafter (C3 and C4, respectively) and (iii) genes which show increase or decrease in expression levels, in particular in late stationary phase at 14, 16 h (C5 and C6, respectively) [Figure 2; Additional file 2].

**Figure 3:**
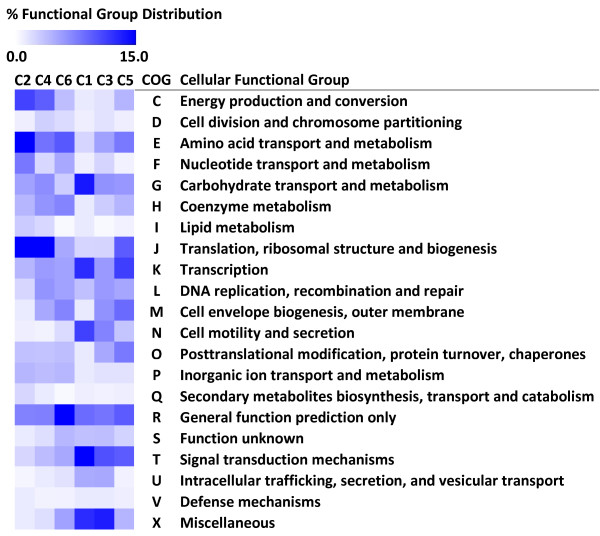
**Functional distribution of differentially expressed genes within clusters**. Calorimetric representation of the percentage distribution of genes, within each of the clusters identified (see Figure 2), across the different Clusters-of-Orthologous-Groups (COG) cellular functional categories. Clusters (C2, C4, C6) and (C1, C3, C5) are clusters in which the genes displayed a decreasing or increasing trend in expression, respectively, in various growth phases during Avicel^® ^fermentation by *Clostridium thermocellum *ATCC 27405.

Genes within each cluster were further classified as per their Clusters-of-Orthologous-Groups (COG) based cellular function and the percentage distribution of genes within each cluster among the different COG categories is shown in Figure 3. The majority of genes in clusters C2, C4 and C6, demonstrated to have moderate to significant decrease in expression in various growth phases during the course of cellulose fermentation, belonged to COG categories with cellular functions related with energy production (COG category C), amino acid (E), nucleotide (F) transport and metabolism, glycolysis (G), coenzyme metabolism (H) and translation, ribosomal structure (J). On the other hand, majority of genes that exhibited increasing trend in gene expression, grouped in clusters C1, C3 and C5, were involved in cellular functions related with cell motility (COG category N; flagellar-, pili-related genes), signal transduction (T), carbohydrate metabolism (G; primarily cellulosome-related genes), transcriptional regulation (K) and DNA recombination including phage-related defense mechanisms (L).

The operon structure prediction for *C. thermocellum *ATCC 27405 by DOOR database ([23]; http://csbl1.bmb.uga.edu/OperonDB/) was used to estimate the correlation for co-regulation of genes in contiguous regions of the genome within predicted operons. Overall there was significant correlation between the total number of genes and the number of genes differentially expressed in a predicted operon that exhibited co-regulated patterns in expression with either concerted increase (9 operons, R-value 0.97) or decrease (30 operons, R-value 0.81-0.96) in transcript levels (data not shown). Examples included two large predicted operons, Cthe0480-0496 (17 ORFs) and Cthe2908-2928 (21 ORFs), in which 14 and 13 genes were differentially expressed, respectively. The former operon, containing several genes involved in flagellar biosynthesis, pili assembly, chemotaxis and signal transduction, displayed an increasing trend in expression while the latter operon, containing genes encoding several large and small ribosomal subunit proteins, showed a progressively decreasing trend in expression over the course of cellulose fermentation.

### Central metabolism and mixed-acid fermentation genes

#### Upstream of phosphoenolpyruvate

**Figure 4:**
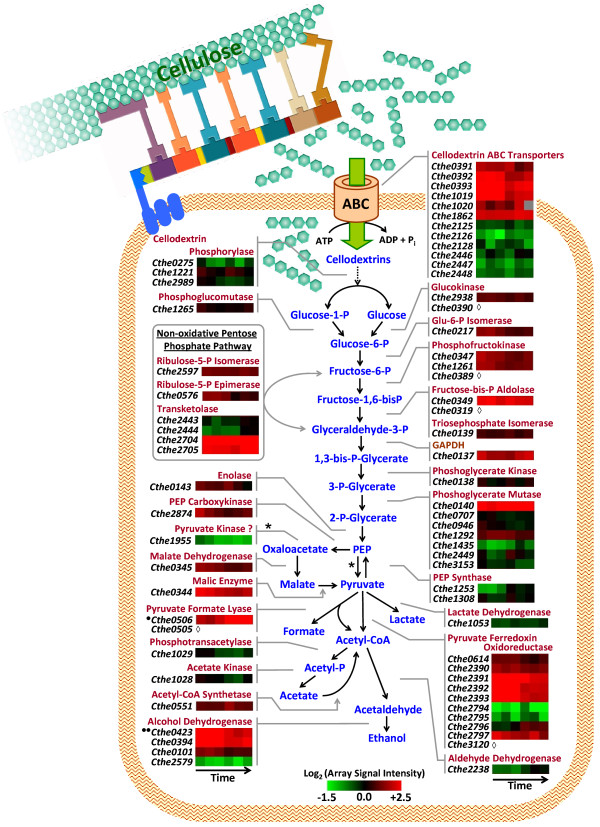
**Expression of genes involved in cellodextrin transport and catabolism during cellulose fermentation**. Schematic representation of cellulose degradation by cell surface attached cellulosomal complex, transport of cellodextrin hydrolysis products into the cell by ABC sugar transporters and intracellular catabolism of glucose to various metabolic end-products. Heat plot representation of transcript expression [as Log_2 _(array signal intensity)] for genes (known and putative) involved in cellodextrin transport and hydrolysis, pentose phosphate pathway, glycolytic conversion of glucose to pyruvate and anaerobic fermentation of pyruvate to organic acids (formate, lactate, acetate) and ethanol, over the course of Avicel^® ^fermentation by *Clostridium thermocellum *ATCC 27405. Cellulosome schematic is an adaptation of the image from the U.S. Department of Energy Genome Programs website image gallery (http://genomics.energy.gov; [40]);***one black circle ***- Cthe0506 is *pfl*-activating enzyme; ***two black circles ***- Cthe0423 encodes a bi-functional acetaldehyde/alcohol dehydrogenase enzyme involved in direct conversion of acetyl-CoA to ethanol; ***open diamond ***- Microarray data is not available.

In general, genes involved in the glycolysis pathway for conversion of glucose-6-phosphate to phosphoenolpyruvate (PEP) either had no change in expression or displayed decreased expression during stationary phase of growth and belonged to clusters C2, C4 and C6 (Figure 4, Additional file 4: Expression of genes upstream of PEP). Both copies of phosphofructokinase (Cthe0347 and Cthe1261), a key regulated enzyme in the Embden-Meyerhoff pathway, showed 1.5-2 fold lowered expression in stationary phase (Figure 4). *C. thermocellum *has multiple homologues of several metabolic enzymes encoded in the genome, including seven copies of phosphoglycerate mutase involved in the conversion of 3-phosphoglycerate to 2-phosphoglycerate. Among these, Cthe0140 had maximal expression throughout the fermentation, Cthe1292 and Cthe0946 displayed regulated expression, while the other four copies displayed relatively minimal expression during cellulose fermentation (Figure 4).

The pentose phosphate pathway is important for production and supply of key intermediates involved in the synthesis of nucleotides and aromatic amino acids. The *C. thermocellum *genome encodes several enzymes in the non-oxidative branch of the Pentose Phosphate (PP) pathway including ribulose-5-P isomerase (Cthe2597) and ribulose-5-P epimerase (Cthe0576) (Figure 4, Additional file 4). During cellulose fermentation, the epimerase gene was downregulated by up to 2-fold in stationary phase, while the isomerase gene was expressed at high levels throughout the course of the fermentation. *C. thermocellum *also has two pairs of contiguous genes encoding transketolases (Cthe2443-44 and Cthe2704-05) which catalyze several reactions in the PP pathway, of which only the Cthe2704-05 pair shows maximal expression during cellulose fermentation (Figure 4). Sequence homology-based annotation has however not revealed a transaldolase in *C. thermocellum*.

#### Downstream of phosphoenolpyruvate

Similar to glycolytic enzymes, a majority of the genes involved in conversion of phosphoenolpyruvate to pyruvate and mixed-acid fermentation of pyruvate to various organic acids and ethanol were downregulated during stationary phase of *C. thermocellum *growth on cellulose (Figure 4, Additional file 5: Expression of genes downstream of PEP). Several Gram-positive organisms, including representatives in the Clostridial species such as *C. phytofermentans *and *C. cellulolyticum*, encode genes for pyruvate phosphate kinase, which catalyzes the conversion of PEP to pyruvate, but genome annotation has not identified a homologous gene in *C. thermocellum*. An ORF recently suggested as a putative pyruvate kinase homologue, Cthe1955 [24], had relatively minimal expression during cellulose fermentation (Figure 4). *C. thermocellum *however has two copies of the gene encoding pyruvate phosphate dikinase (Cthe1253 and Cthe1308), which catalyzes the reverse reaction for conversion of pyruvate to PEP. This enzyme is suggested to play an anabolic role in gluconeogenesis and consistent with this, Cthe1253 is upregulated in stationary phase during growth on cellulose. Sparling and colleagues have proposed an alternate route for conversion of PEP to pyruvate *via *oxaloacetate and malate (Figure 4; Sparling, *personal communication*). Genes encoding all three enzymes in this alternative route, the gluconeogenic PEP carboxykinase (Cthe2874), malate dehydrogenase (Cthe0345), and malic enzyme (Cthe0344), were expressed at high levels, suggesting that this putative pathway is active in *C. thermocellum *during growth on cellulose.

*C. thermocellum *contains two clusters of genes (Cthe2390-2393 and Cthe2794-2797) encoding the gamma, delta, alpha and beta subunits, respectively, of the thiamine-pyrophosphate (TPP) dependent pyruvate ferredoxin oxidoreductase (POR) which catalyzes the oxidation of pyruvate to acetyl-CoA. While all the genes in the Cthe2390-2393 cluster displayed maximal expression during cellulose fermentation, only a single gene in the Cthe2794-2797 cluster, encoding the TPP-binding beta subunit of the POR protein complex (Cthe2797), had high transcript levels which decreased in stationary phase (Figure 4). This is in contrast to studies by Sparling and colleagues who reported expression of Cthe2794-97 transcripts by RT-PCR during log phase of growth on alpha-cellulose with only weak expression of the Cthe2390-93 cluster [13]. However, the observed trends in gene expression may be due to differences in culture conditions between the two studies. While qPCR analysis was done with batch cultures in Balch tubes with no pH control [13], microarray analysis in this study was conducted with samples from controlled fermentations in bioreactors with pH control.

#### Mixed-acid fermentation

*C. thermocellum *encodes several genes involved in the mixed-acid fermentation pathways for conversion of pyruvate to organic acids (lactate, formate, acetate) and ethanol (Additional file 5, Expression of genes downstream of PEP). These include two putative lactate dehydrogenase genes (*ldh*; Cthe0345, Cthe1053) involved in conversion of pyruvate to lactate. Previous studies have reported detecting LDH activity in cell extracts of *C. thermocellum *[14,25,26] and RT-PCR analysis has shown expression of both *ldh *genes during cellulose batch and continuous fermentations [11,13]. LDH, Cthe1053, cloned and expressed in *E. coli*, has been functionally characterized and demonstrated to oxidize pyruvate to lactate [27]. In this study, the Cthe1053 gene displayed low expression and lactate was not detected during cellulose fermentation. Although another gene annotated as *ldh *(Cthe0345) was expressed at high levels, this may be related to the participation of the encoded enzyme as a malate dehydrogenase in the alternate route for conversion of PEP to pyruvate, as discussed earlier (Figure 4). Pyruvate formate lyase (*pfl*) catalyzes the conversion of pyruvate to formate, along with the formation of acetyl-CoA. Sparling *et al*, reported formate synthesis in *C. thermocellum via *this pathway with detection of transcripts for *pfl *(Cthe0505) and the *pfl *activating enzyme (Cthe0506) by RT-PCR [13]. In this study, two out of four putative *pfl *activating enzymes (Cthe0506, Cthe0647) were expressed at relatively high levels during cellulose fermentation (Additional file 5; data not available for *pfl*, Cthe0505). However, formate was not detectable in the culture supernatant consistent with other previous reports [25,28].

Acetyl-CoA is further catabolized to acetate with generation of ATP or to ethanol with reoxidation of NADH. *C. thermocellum *encodes an NADH-dependent aldehyde dehydrogenase (*aldH*, Cthe2238), which catalyzes the conversion of acetyl-CoA to acetaldehyde, and several iron-containing alcohol dehydrogenases (Cthe0101, Cthe0394 [*adhY*] and Cthe2579 [*adhZ*]) for alcohol synthesis from acetaldehyde; also encoded is a bi-functional acetaldehyde/alcohol dehydrogenase (Cthe0423, *adhE*) which catalyzes the direct conversion of acetyl-CoA to ethanol (Figure 4). AdhE has been proposed to be a key enzyme for ethanol synthesis in *C. thermocellum *and transcription of *adhE*, *adhY *and *adhZ *has been confirmed by RT-PCR analysis in cellobiose and cellulose cultures of *C. thermocellum *[11,19]. In this study, the *aldH *gene showed increased expression during stationary phase while the three *adh *genes, Cthe0394, Che0423 and Cthe0101, were actively expressed during cellulose batch fermentation with the latter showing decreased expression in stationary phase (Figure 4, Additional file 5).

Acetyl-CoA is indirectly converted to acetate *via *acetyl-phosphate through the action of two enzymes, encoded by the contiguous genes, phosphotransacetylase (*pta*, Cthe1029) and acetate kinase (*ack*, Cthe1028), with the generation of one ATP per acetate molecule. The reverse reaction for direct conversion of acetate to acetyl-CoA utilizes ATP and is catalyzed by acetyl-CoA synthetase (*acs*, Cthe0551). Previous studies have confirmed the expression of acetate kinase through RT-PCR [11] and enzyme activity measurements [25]. In this study, both *pta *and *ack *genes were expressed at low levels which further decreased in stationary phase; whereas, the *acs *gene was expressed at relatively higher levels over the entire course of the fermentation (Figure 4, Additional file 5). The observed gene expression patterns are consistent with our metabolite analysis of the fermentation medium, which showed decreasing acetate-to-ethanol molar ratios during entry into stationary phase (Figure 1). Several genes involved in conversion of pyruvate to other intermediate metabolites such as α-ketoglutarate, which is a building block for amino acid and nucleic acid biosynthesis, also showed high level of expression during active growth but lowered levels in stationary phase (Additional file 5), possibly due to reduced metabolic need under slow growth and nutrient-limited conditions.

### Energy generation and redox balance

Overall, the genes involved in maintaining the intracellular redox conditions and cellular energy production systems belonged to clusters C2, C4 and C6 and were downregulated with decreasing growth rate over the course of cellulose batch fermentation (Additional file 6, Expression of genes involved with energy generation and redox balance).

**Figure 5:**
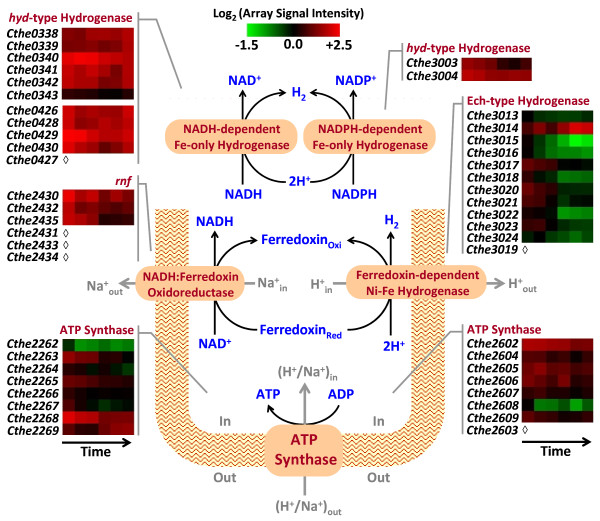
**Expression of genes involved in maintaining cellular REDOX status**. Heat plot representation of transcript expression [as Log_2 _(array signal intensity)] for genes (known and putative) involved in cellular reduction and oxidation reactions, co-factor recycling, hydrogen production and ATP synthesis, over the course of Avicel^® ^fermentation by *Clostridium thermocellum *ATCC 27405; ***open diamond ***- Microarray data is not available.

*C. thermocellum *uses the hydrogenase-mediated pathway for production of molecular hydrogen to dispose the excess reducing equivalents generated during carbohydrate catabolism. Putative hydrogenases encoded in the *C. thermocellum *genome include, (i) Ferredoxin-dependent Ech-type NiFe-hydrogenase (Cthe3013-3024), (ii) two NADH-dependent Fe-only hydrogenases (Cthe0338-0343 and Cthe0426-0430) and (iii) NADPH-dependent Fe-only hydrogenase (Cthe3003-3004) [13,14]. Ech hydrogenase and NADH:Ferredoxin oxidoreductase (*rnf*, Cthe2430-2435) complexes reoxidize the ferredoxin reduced during POR catalyzed conversion of pyruvate to acetyl-CoA (Figure 5). In the process, the complexes pump H+/Na+ ions across the cell membrane and create proton gradients for powering ATP synthesis by ATP synthase and H+/Na+ transporting ATPase complexes encoded in genomic regions, Cthe2602-2609 and Cthe2262-2269, respectively. Carera *et al*. [13] demonstrated transcription of representative genes in these hydrogenase complexes using RT-PCR and Rydzak *et al*. [14] reported detecting activities from all three classes of hydrogenases during growth on cellobiose. In this study, we observed significant expression of genes encoding NADH-, and NADPH-dependent hydrogenases and relatively lower expression of Ech hydrogenase during active growth phase of cellulose fermentation. Expression of hydrogenase and ATP synthase genes was downregulated by up to 2.5-fold in stationary phase with the exception of the *hypD *(Cthe3014) gene, encoding the hydrogenase formation protein, which exhibited a 3-fold increase in expression (Figure 5; Additional file 6). Genes involved in maintaining cellular reduction-oxidation status have been demonstrated to be important metabolic engineering targets for increasing solvent yields in thermophilic anaerobes [29]. A recent genome-scale metabolic model of *C. thermocellum *predicted a 15-fold increase in maximum ethanol production resulting from deletion of hydrogenase gene, Cthe3003 [24].

### Cellulosomal and non-cellulosomal carbohydrate active enzymes

In *C. thermocellum*, cellulases and other polysaccharide degrading enzymes are assembled together in large protein complexes, termed the cellulosome, on the cell-surface. The cellulosome complex has a primary scaffoldin protein, CipA, containing 9 type-I cohesin-modules and catalytic subunits, each containing a complementary type-I dockerin module, interact strongly with the cohesin module for assembly onto the scaffoldin. CipA with bound enzymes is in turn attached to the cell surface *via *interaction between the CipA-borne type-II dockerin and type-II cohesins of the cell wall anchor proteins. During growth on insoluble substrates, the cells are tightly attached to the substrate *via *the carbohydrate binding module (CBM) borne by CipA and many catalytic subunits of the cellulosomes forming a cell-cellulosome-carbohydrate complex.

**Figure 6:**
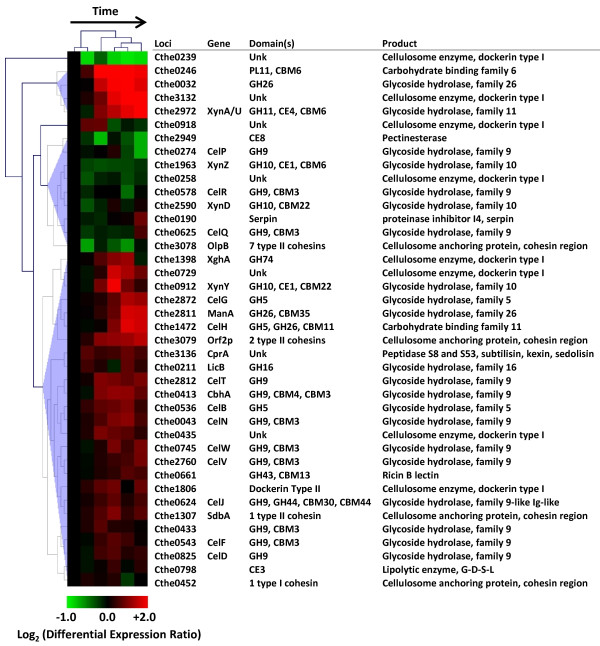
**Cellulosomal genes differentially expressed during cellulose fermentation**. Heat plot representation of Log_2 _(Differential Expression Ratio) and hierarchical clustering of cellulosomal genes showing statistically significant differences in transcript expression over the course of Avicel^® ^fermentation by *Clostridium thermocellum *ATCC 27405. Domain key: GH = Glycoside Hydrolase, CE = Carbohydrate Esterase, PL = Polysaccharide Lyase, CBM = Carbohydrate Binding Module, Unk = unknown, based on the Carbohydrate Active Enzymes database (http://www.cazy.org; [41])

*C. thermocellum *genome has revealed the presence of more than 70 catalytic subunits containing type-I dockerin and 8 non-catalytic structural components ([30]; Additional file 7, Expression of cellulosomal and non-cellulosomal CAZyme genes). Recent studies have provided evidence for the functional expression of more than 65 cellulosome components in *C. thermocellum *at the protein level. Quantitative proteomic analysis of cellulosomes isolated from *C. thermocellum *cultures grown on different carbon sources revealed a substrate-dependent regulation of catalytic subunit distribution in cellulosomes [16,31]. In this study, during growth of *C. thermocellum *on crystalline cellulose, a temporally regulated pattern of changes in cellulosomal composition was observed at the transcript level (Figure 6, Additional file 7). Among 20 catalytic subunit genes with the highest expression at transcript-level (this study) and protein-level (previous study, [16]), 12 genes were common suggesting significant correlation between the two measurements (data not shown). Cellulosomal and other CAZyme genes were primarily grouped in clusters C1, C3 and C5 which showed upregulated expression during different phases of cellulose fermentation (Figures 2, 3).

Structural cellulosome components include the scaffoldin CipA (Cthe3077) and seven anchor proteins, five containing type-II cohesins (Cthe1307/SdbA, Cthe 3078/OlpB, Cthe3079/Orf2p, Cthe0735 and Cthe0736) and two containing type-I cohesin (Cthe3080/OlpA, Cthe0452). Among these, genes encoding CipA, Orf2p, OlpB and OlpA exhibited maximal expression during cellulose fermentation (Additional file 7). Expression of *orf2p *increased by up to 2-fold over the course of the batch fermentation in agreement with Dror *et al*. who reported an inverse correlation between growth rate and mRNA levels of the anchor genes, *olpB*, *orf2p *and the scaffoldin *cipA *[8]. However, in this study, expression levels of *cipA *did not change significantly during batch growth and *olpB *displayed a moderate decrease in expression in stationary phase (Figure 6, Additional file 7).

**Figure 7:**
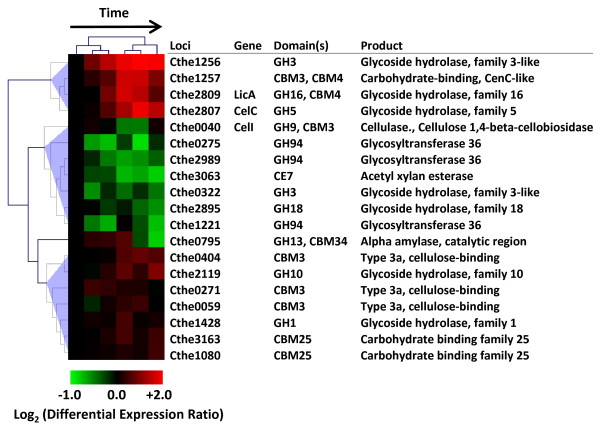
**Non-cellulosomal genes differentially expressed during cellulose fermentation**. Heat plot representation of Log_2 _(Differential Expression Ratio) and hierarchical clustering of non-cellulosomal CAZyme genes showing statistically significant differences in transcript expression over the course of Avicel^® ^fermentation by *Clostridium thermocellum *ATCC 27405. Domain key: GH = Glycoside Hydrolase, CE = Carbohydrate Esterase, PL = Polysaccharide Lyase, CBM = Carbohydrate Binding Module, Unk = unknown, based on the Carbohydrate Active Enzymes database (http://www.cazy.org; [41])

Catalytic cellulosome subunits display a wide range of hydrolytic capabilities including endo-, exo-glucanases, hemicellulases, and pectinases, among other enzymatic activities [3]. Hierarchical clustering of differentially expressed genes revealed increased expression of several catalytic components over the course of cellulose fermentation (Figure 6). In agreement with an earlier study reporting a growth rate dependent regulation of the endoglucanases belonging to GH5 (*celB*, *celG*) and GH9 (*cel*D) families [9], expression of these genes increased with decreasing growth rate, with peak expression at 12 or 14h into the fermentation. However, the *celS *GH48 family processive exoglucanase, also reported to be growth-rate regulated [7], showed a statistically insignificant increase in expression over time (Figure 6, Additional file 7). In addition to the cellulosomal enzymes, *C. thermocellum *genome encodes sequences for 35 non-cellulosomal CAZymes (no dockerin domain; Additional file 7), which were also differentially expressed during cellulose fermentation (Figure 7). For example, members of the GH94 family, involved in intracellular phosphorolytic cleavage of cellodextrin and cellobiose, were downregulated as substrate availability decreased over the course of the fermentation. Whereas, two non-cellulosomal enzymes encoded by contiguous genes, Cthe1256-1257, exhibited increased expression by up to 4-fold in stationary phase.

Newcomb and Wu [32] characterized a LacI family transcriptional regulator, GlyR3 (Cthe2808), in *C. thermocellum *that was shown to regulate the expression of two non-cellulosomal CAZymes, a GH16 family lichinase (*licA*, Cthe2809) and a GH5 family cellulase (*celC*, Cthe2807), all encoded together in the putative *celC *operon, Cthe2807-2809. During cellulose fermentation, genes in this operon displayed relatively little expression in exponential phase but their transcript levels continually increased with maximal expression of >3-fold in stationary phase (Figure 7, Additional file 7). Mishra *et al*. also observed a similar expression pattern during cellobiose fermentation in which *celC *transcripts were detected exclusively in early stationary phase after cessation of growth [10]. Differential expression of the operon in the absence of laminaribiose, the identified GlyR3 inducer [32], suggests that other cellulose-derived oligosaccharides may also act as inducers or other regulatory mechanisms may be involved.

Recent evidence suggests the possible role of membrane-associated anti-sigma factors in extracellular carbohydrate-sensing and CAZyme gene regulation in *C. thermocellum*. Kahel-Raifer *et al*. identified several putative bicistronic operons in the *C. thermocellum *genome, each operon encoding an RsgI-like anti-σ factor and a putative alternative sigma factor σ^I ^(SigI) and proposed a regulatory model, wherein RsgI senses the presence of biomass components in the extracellular medium via its CBM domain while SigI mediates the intracellular activation of appropriate CAZyme genes that are necessary for hydrolysis of the polysaccharide substrate, in response to the transmitted signal [33]. In this study, three of the σ^I ^encoding genes (Cthe0058, Cthe0268, Cthe0403) that are associated with anti-σ^I ^-like genes bearing cellulose-binding CBM3 domains were all upregulated, with Cthe0268 showing ~5-fold increased expression, during later stages of the cellulose fermentation (Additional file 8: Expression of genes involved in carbohydrate sensing and CAZyme regulation). The observed pattern in expression of CBM3-related σ^I ^genes, i.e., their increased expression in stationary phase, seems to differ from the regulatory model proposed by Kahel-Raifer *et al*., who suggested induced expression of sigma factor in the presence of the polysaccharide substrate [33]. This is probably explained by the presence of residual Avicel in the stationary phase or perhaps suggests the involvement of additional mechanisms, such as growth rate, in the regulation of *sigI *genes. However, several genes encoding GH9 family cellulases (Cthe0043/CelN, Cthe0413/CbhA, Cthe0543/CelF, Cthe0745/CelW, Cthe2812/CelT etc.) were also upregulated with peak expression in early-to-late stationary phase (Additional file 7) and are potentially part of SigI regulon in *C. thermocellum*.

Several of the cellulosomal CAZymes with significant upregulated expression in later stages of fermentation belonged to GH families with hydrolytic capabilities specializing in degradation of non-cellulolytic substrates - for example, GH26 family mannanases (Cthe0032, *manA*/Cthe2811), GH74 family xyloglucanase (*xghA*/Cthe1398), Family 11 pectate lyase (Cthe0246), GH10 and GH11 family xylanases (*xynY*/Cthe0912, *xynA-U*/Cthe2972), bi-functional CelH (Cthe1472) containing GH5 and GH26 domains etc. (Figure 6, Additional file 7). Given that *C. thermocellum *releases the cellulosomes in stationary phase [34], it is likely that the increased expression of non-cellulolytic GH family enzymes during the latter part of growth is aimed towards enriching this population of enzymes in the free cellulosomes to aid in exposing the preferred substrate of cellulose from untapped resources in the cellular vicinity. Increase in expression of the two serine protease inhibitor components (Cthe0190, Cthe0191) may serve to protect the free cellulosomes from proteolytic degradation.

### Cellodextrin transport-related genes

Ten percent of the ORFs in the *C. thermocellum *genome encode proteins that are involved in transport of oligosaccharides, amino acids, inorganic and metal ions, co-factors etc. *C. thermocellum *has been reported to use ABC-type systems for transport of oligosaccharides derived from cellulose hydrolysis [35]. Recently, Shoham and colleagues characterized several ABC sugar binding proteins in *C. thermocellum *(CbpA, Cthe0393; CbpB, Cthe1020; CbpC, Cthe2128; CbpD, Cthe2446) based on their affinity to glucose and G2-G5 cello-oligosaccharides [36]. In this study, genes in contiguous regions (Cthe0391-0393 and Cthe1019-1020) encoding CbpA and CbpB proteins with binding affinities to G3 and G2-G5 beta-1,4-glycans, respectively, and Cthe1862, encoding another sugar binding protein of unknown specificity, were expressed at high levels throughout the course of cellulose fermentation (Figure 4). This observation is consistent with the study by Zhang and Lynd demonstrating the preference of *C. thermocellum *for importing 4-glucose-unit chains during growth on cellulose. The bioenergetic implications of importing long cellodextrins are two-fold, (i) from reduced cost of transport as only one-ATP molecule is needed per transport event irrespective of the chain length and (ii) additional energetic advantage from phosphorolytic cleavage of the imported oligosaccharides [37].

### Chemotaxis, signal transduction and motility genes

The majority of genes involved in flagellar- and pili-based cell motility and chemotaxis-based signal transduction mechanisms displayed an increasing trend in expression over the course of cellulose fermentation. Approximately, 81% of all differentially expressed (DE) genes belonging to COG category N (motility-related) and 64% of all DE genes belonging to COG category T (signal transduction) were grouped to clusters C1, C3 and C5, which contain genes showing increased expression in various stages of growth (Figures 2, 3). In *C. thermocellum*, many of the genes involved in flagellar biosynthesis/assembly and two-component signal transduction are clustered in a genomic region containing ORFs ranging from Cthe0462-0496. Several genes in this region, within putative operons Cthe0462-0464 (all 3 genes) and Cthe0480-0496 (14 out of 17 genes), were coordinately upregulated during cellulose fermentation. Many genes in another genomic region, Cthe1100-1107, encoding fimbrial assembly and type II secretion system proteins, also showed increased expression by up to 3-fold during growth. These results suggest potentially increased motility of *C. thermocellum *during later stages of the fermentation. This is in contrast to reports of decreased expression of flagellar and chemotaxis genes in solventogenic members of the clostridia, *C. beijerinckii *[38] and *C. acetobutylicum *[39] during shift from acidogenic to solventogenic phase or at the onset of sporulation, respectively. In *C. thermocellum*, upregulated expression of motility- and chemotaxis-related genes under conditions of low substrate availability, suggest a cellular strategy oriented towards enhancing the ability of cells to sense the environment and appropriately respond to the ambient signals through activation of the cellular motility systems.

## Conclusions

Due to its native cellulolytic capability and ability to ferment cellulose hydrolysis products directly to ethanol, *Clostridium thermocellum *is an attractive candidate microorganism for consolidate bioprocessing of plant biomass to biofuels. Understanding the microbial physiology associated with cellulase synthesis, cellulose degradation, and cellular growth is vital to identifying genetic targets for manipulation and strain improvement. In this study, we probed *C. thermocellum *gene expression during the course of cellulose fermentation using whole genome microarray technology. Time course analysis of gene expression coupled with clustering of genes with similar temporal patterns in expression revealed an overall decrease in metabolic potential of the organism over the course of the fermentation. Several genes involved in energy production, translation, glycolysis and amino acid, nucleotide and coenzyme metabolism displayed a progressively decreasing trend in gene expression. In comparison, genes involved in cell structure and motility, chemotaxis, signal transduction, transcription and cellulosomal genes displayed an increasing trend in gene expression.

While growth-rate related changes in cell growth and metabolism genes have been well documented, the increasing trend in expression of CAZyme genes, especially when the overall energy and protein synthesis capacity of the cells is at its minimal throughput in the stationary phase is rather surprising. This might denote a cellular strategy to channel the available resources towards the cellulolytic machinery, thereby increasing its chances of finding new sources of nutrition. During active growth, the cellulosome complex is tightly adhered to the cell surface and also to the solid substrate forming a tri-complex between cells, cellulosome and cellulose. But, when growth begins to slow-down, *C. thermocellum *is known to release the cellulosomes into the culture medium [34], perhaps through sensing the decreasing supply of oligosaccharides. The released cellulosomes could then act as 'deployed soldiers in the battlefield,' whereby they are free to diffuse and 'hunt' for alternate sources of nutrients in the environment. Increasing the expression of non-cellulolytic enzymes and thus modulating the composition of the released cellulosomes would enhance the chances for successfully 'un-wrapping' the preferred substrate of cellulose from other plant polysaccharides such as hemicellulose and pectin. However, it is not yet known if there are distinct differences in the composition of the attached *vs *the detached cellulosomes in *C. thermocellum *and warrants further study.

In conjunction with changes in potential composition of cellulosome and its release, increase in motility and signal transduction capability of the cells in stationary phase further highlights the evolution of this organism to feast and famine conditions in nature. If we assume that the cells release the cellulosomes in search of alternate nutrient sources, then it would be advantageous to correspondingly enhance the cells' ability to sense the oligomeric degradation products resulting from the activity of cellulosomes, although such mechanisms are currently unknown in this organism. Similarly, altering gene expression to improve cellular motility systems would help in appropriately orienting the cells' movement towards the nutrient gradient of interest. Hence the observed increase in expression of flagellar genes and chemotaxis genes is likely linked to adaptation and survival under famine conditions. Relatively little is understood about nutrient sensing mechanisms and the genes that are regulated in response to such senses in *C. thermocellum*. To our knowledge, this is the first global whole cell gene expression study in *C. thermocellum*, which enhances the current understanding of *C. thermocellum *physiological changes during cellulose fermentation and also lays the foundation for future studies with natural biomass.

## Authors' contributions

BR, SDB and JRM conceived and designed the study; CKM carried out the growth studies; MR carried out the metabolite analysis; BR conducted the fermentations, carried out the microarray studies, statistical data analysis and drafted the manuscript with input from JRM and SDB. All authors read and approved the final manuscript.

## Supplementary Material

Additional file 1 **RT-qPCR validation of microarray results**. Comparison of gene expression ratios estimated by microarray hybridization and RT-qPCR for five representative genes across two different time-points. Click here for file

Additional file 2 **Functionally annotated genes differentially expressed during cellulose fermentation**. Microarray expression data for functionally annotated genes differentially expressed in time-course analysis of transcript level changes during Avicel^® ^fermentation by *Clostridium thermocellum *ATCC 27405. Click here for file

Additional file 3 **Hypothetical, unknown genes differentially expressed during cellulose fermentation**. Microarray expression data for hypothetical, unknown function genes differentially expressed in time-course analysis of transcript level changes during Avicel^® ^fermentation by *Clostridium thermocellum *ATCC 27405. Click here for file

Additional file 4 **Expression of genes upstream of phosphoenolpyruvate**. Microarray expression data for genes involved in the glycolysis pathway for conversion of glucose-6-phosphate to phosphoenolpyruvate during Avicel^® ^fermentation by *Clostridium thermocellum *ATCC 27405. Click here for file

Additional file 5 **Expression of genes downstream of phosphoenolpyruvate**. Microarray expression data for genes involved in conversion of phosphoenolpyruvate to pyruvate, and mixed-acid fermentation of pyruvate to various organic acids and ethanol, during Avicel^® ^fermentation by *Clostridium thermocellum *ATCC 27405. Click here for file

Additional file 6 **Expression of genes involved with energy generation and redox balance **Microarray expression data for genes involved in maintaining the intracellular redox conditions and cellular energy production systems during Avicel^® ^fermentation by *Clostridium thermocellum *ATCC 27405. Click here for file

Additional file 7 **Expression of cellulosomal and non-cellulosomal CAZyme genes **Microarray expression data for genes encoding cellulosomal and non-cellulosomal carbohydrate active enzymes during Avicel^® ^fermentation by *Clostridium thermocellum *ATCC 27405. Click here for file

Additional file 8 **Expression of genes involved in carbohydrate sensing and CAZyme regulation **Microarray expression data for genes involved in extracellular carbohydrate-sensing and regulation of carbohydrate active enzymes during Avicel^® ^fermentation by *Clostridium thermocellum *ATCC 27405. Click here for file
