# Supplementary material for: Transcriptomic analysis of Clostridium thermocellum ATCC 27405 cellulose fermentation
Source: BMC Microbiol. 2011 Jun 14;11:134. doi: 10.1186/1471-2180-11-134 (PMC3130646; doi:10.1186/1471-2180-11-134)
Supplement: Additional file 1 — RT-qPCR validation of microarray results. Comparison of gene expression ratios estimated by microarray hybridization and RT-qPCR for five representative genes across two different time-points. [file 1471-2180-11-134-S1.DOC]

**Additional file 1: RT-qPCR validation of microarray results**

Comparison of gene expression ratios estimated by microarray hybridization and RT-qPCR for five representative genes across two different time-points.
